# No Evidence of Re-infection or Person-to-Person Transmission in Cured COVID-19 Patients in Guangzhou, a Retrospective Observational Study

**DOI:** 10.3389/fmed.2020.593133

**Published:** 2020-11-30

**Authors:** Gang Xu, Feng Liu, Min Ye, Jun Zhao, Qing Li, Congrui Feng, Yudong Hu, Yueping Li, Haiyan Shi, Fuchun Zhang, Yuwei Tong, Wei Ma

**Affiliations:** ^1^Department of Geriatric Medicine, Guangzhou First People's Hospital, School of Medicine, South China University of Technology, Guangzhou, China; ^2^Department of Respiratory Medicine, Guangzhou Eighth People's Hospital, Guangzhou, China

**Keywords:** COVID-19, cured patients, re-infection, person-to-person transmission, antibody

## Abstract

**Objectives:** To clarify the clinical characteristics of cured patients with coronavirus disease (COVID-19), and to clarify the re-infection and person-to-person transmission in the cured.

**Methods:** A total of 187 cured COVID-19 patients with antibody test were followed up every 2 weeks in this retrospective observational study. Assessment for general condition, symptoms, epidemiological contact history, polymerase chain reaction (PCR) assay, and antibody tests were performed and recorded. Information from Guangzhou CDC was also screened.

**Results:** There were 33 (17.6%) patients with negative results for IgG and 35 (18.7%) patients with positive results for IgM. The average days of antibody detection from disease onset were 53.0. PCR assay was positive in 10 (5.3%) patients during the follow-up. Neither IgG nor IgM results showed a relationship with PCR test results (all *P* > 0.05). Neither re-infection nor person-to-person transmission was found in the cured patients. Factors associated with appearance of antibody comprised hospitalization days (OR: 1.06, 95%CI: 1.02–1.11, *P* = 0.006) and antibiotics treatment (OR: 3.50, 95%CI: 1.40–8.77, *P* = 0.007).

**Conclusions:** In our study, no evidence of person-to-person transmission was found in cured COVID-19 patients. There seemed to be no re-infection in the cured COVID-19 patients in Guangzhou. These finding suggest that the cured do not cause the spread of disease. Additionally, neither IgG nor IgM can be used to replace the PCR test in cured patients.

## Introduction

Coronavirus disease (COVID-19) is an acute infectious disease caused by the severe acute respiratory syndrome coronavirus 2 (SARS-CoV-2), and is characterized by high morbidity and mortality (1, 2). COVID-19 outbreak began in China in December 2019 and spread rapidly worldwide, with the World Health Organization declaring it a pandemic on March 11, 2020. At present, 4,000,000 confirmed cases of COVID-19 have been detected in more than 200 countries, resulting in more than 280,000 deaths (3), and additional patients with COVID-19 are expected to be cured and discharged over time. Prevention remains the focus for control of COVID-19 (4), but the cured or recovered patients should not be ignored. Currently, little is known about cured COVID-19 patients, and there are no studies to clarify the infectious of the cured or guidelines regarding the management of these patients. However, it is very important to understand the clinical characteristics of cured patients, especially with respect to re-infection and person-to-person transmission.

During the immune response activated by the infection, IgM levels are usually elevated earlier, indicating recent infection and infectivity, while elevated IgG levels indicate adaptive immunity (5). However, in patients with COVID-19, the relevance of IgM and IgG antibodies has not been clarified. Researches demonstrated that IgM and IgG could be identified during the middle and later stage of COVID-19, and thus could have a high diagnostic value in patients with acute infection (6–8). Compared with real-time reverse transcriptase polymerase chain reaction (RT-PCR), the detection of antibodies by ELISA is faster, less expensive, and easier to perform. Therefore, antibody detection might be widely used to assist in the diagnosis of SARS-CoV-2 infection. Till date, no study has evaluated the clinical significance of IgM and IgG detection in terms of re-infection and person-to-person transmission, especially in COVID-19 patients who were cured and discharged home.

In this retrospective observational study, we investigated the clinical significance of IgM and IgG in cured patients after SARS-CoV-2 infection. Furthermore, we clarified the re-infection risk and reported person-to-person transmission of the cured patients. We expect that a deeper understanding the characteristics of cured patients with COVID-19 would be of great value in preventing the spread of the disease.

## Methods

This retrospective observational study was conducted from January 20 to March 10, with follow up till April 10, 2020. All cured adult patients with COVID-19 who performed antibody test were enrolled in our study. Patients were followed up in Guangzhou Eighth People's hospital, a government-designated hospital which admitted nearly 80% of the COVID-19 cases in Guangzhou, the capital city of Guangdong Province in southern China. This study was approved by the ethics committee of the Guangzhou Eighth People's Hospital. Because of the retrospective nature of the study design and the grim scenario of COVID-19 pandemic, the Ethics Committee assented to exempt of all informed consents.

### Definition

**COVID-19** was diagnosed as per the World Health Organization's interim guidelines (9). High throughput sequencing or RT-PCR were only performed in subjects with the following features: 1. with a confirmed or suspected contact history of COVID-19; 2. presented with symptoms; 3. with abnormal chest computed tomography (CT) imaging related to COVID-19. A positive result on high throughput sequencing or RT-PCR assay together with at least two of the above three clinical features, confirmed the diagnosis of COVID-19. **Criteria for cured** and discharged to home were as follows: vital signs were stable for more than 3 days; the PCR test was negative two times consecutively 24 h apart; and the acute exudative lung lesions were absorbed or cured on chest CT. **Re-infection** criteria were as follows: typical clinical symptoms; chest CT indicative of new infiltration; and two positive repeat PCR tests performed consecutively at an interval of more than 24 h. All confirmed re-infection cases were reviewed by two senior COVID-19 experts. **Person-to-person transmission** criteria were as follows: The cured were supposed to be the carriers. New confirmed COVID-19 cases occurred after one with unprotected exposure to the cured within 2 weeks. Since all new confirmed COVID-19 cases in Guangzhou were reported to Guangzhou CDC, and Guangzhou CDC released the cases including the exposure to source of transmission daily, the person-to-person transmission was assessed from the reports of CDC.

### Follow Up

All recovered or cured patients with COVID-19 were quarantined at home for 2 weeks after being discharged. They were free to go anywhere in Guangzhou after 2 weeks. The cured patients were followed up every 2 weeks. Follow-up consisted of assessing the general condition, symptoms, living area, PCR assay, and antibody test. Additionally, these recovered patients were required to report if people close to them had been diagnosed with COVID-19. For patients with a positive PCR test, a chest CT was performed immediately, and PCR test was re-performed consecutively at an interval of more than 24 h. The PCR assay and antibody test were performed on the same day. If positive, IgM antibody test would be repeated within 2 weeks. During the study, the researchers screened the report from CDC in Guangzhou every day to determine whether there were any new confirmed COVID-19 cases linked to transmission by the cured patients.

### IgM and IgG Testing

The serum SARS-CoV-2 antibodies (IgM and IgG) were detected using colloidal Gold-based Immunoassays (Colloidal gold kits, Livzon Inc, Zhuhai, China). First, the kit was removed and kept for 30 min at room temperature. Subsequently, 10 μl of plasma sample and 20 μl of whole blood sample were added into the reaction pore until the liquid was fully absorbed. Lastly, two drops of sample diluents were added into the reaction hole until the liquid was fully absorbed. The result could be read in 15 min.

### Statistical Analyses

Shapiro-Wilk normality test was used to assess for normal distribution of data. Continuous variables with normal distribution were expressed as mean ± standard deviations (SD), while those with non-normal distribution were expresses as median and inter quartile range (IQR). Categorical variables were summarized as counts and percentages. For continuous variables, Independent *t*-test or Wilcoxon rank sum test were used. For comparison of categorical variables, Chi-square test and Fisher's exact test were used. Logistic regression analyses were performed to examine the relationship between independent variables and presence of IgG. Determinants with a *P*-value of 0.10 or less in univariate models were initially included in the multivariate model and were then discarded using backward selection. A *P*-values < 0.05 means statistically significant. All data were processed with SPSS version 22.0 for Windows (SPSS, Chicago, IL, USA).

## Results

**Figure 1:**
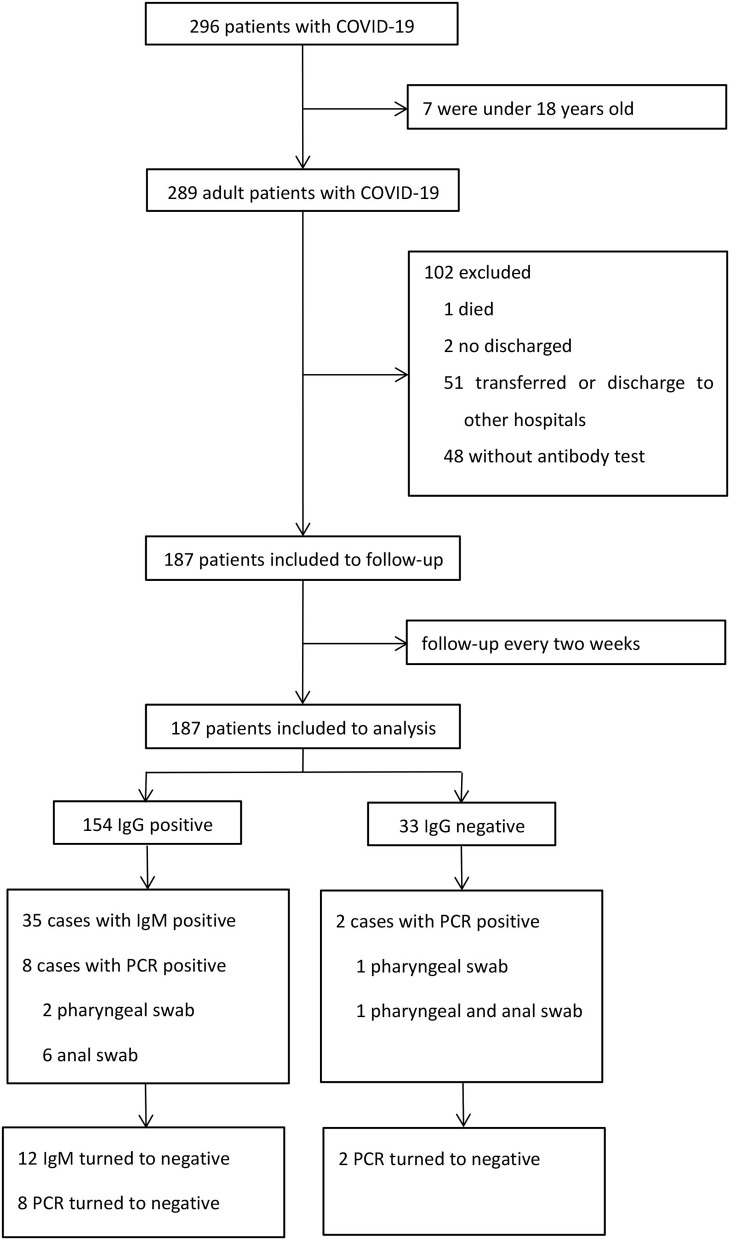
Flow of patients through the study.

A total of 296 patients were diagnosed with COVID-19 from January 20, 2020 to March 10, 2020. Among these patients, one died, two were still hospitalized, seven were under 18 years old, 48 refused to perform antibody test, and 51 were transferred or discharged to other hospitals for treatment (Figure 1). Altogether, 187 patients were screened and followed up at least once in our hospital and subsequently followed up till April 10, and they were included in the final analysis. The mean follow-up time was 45.7 days. No re-infection occurred in any patient after discharge and no medical staffs were infected during the treatment.

**Figure 2:**
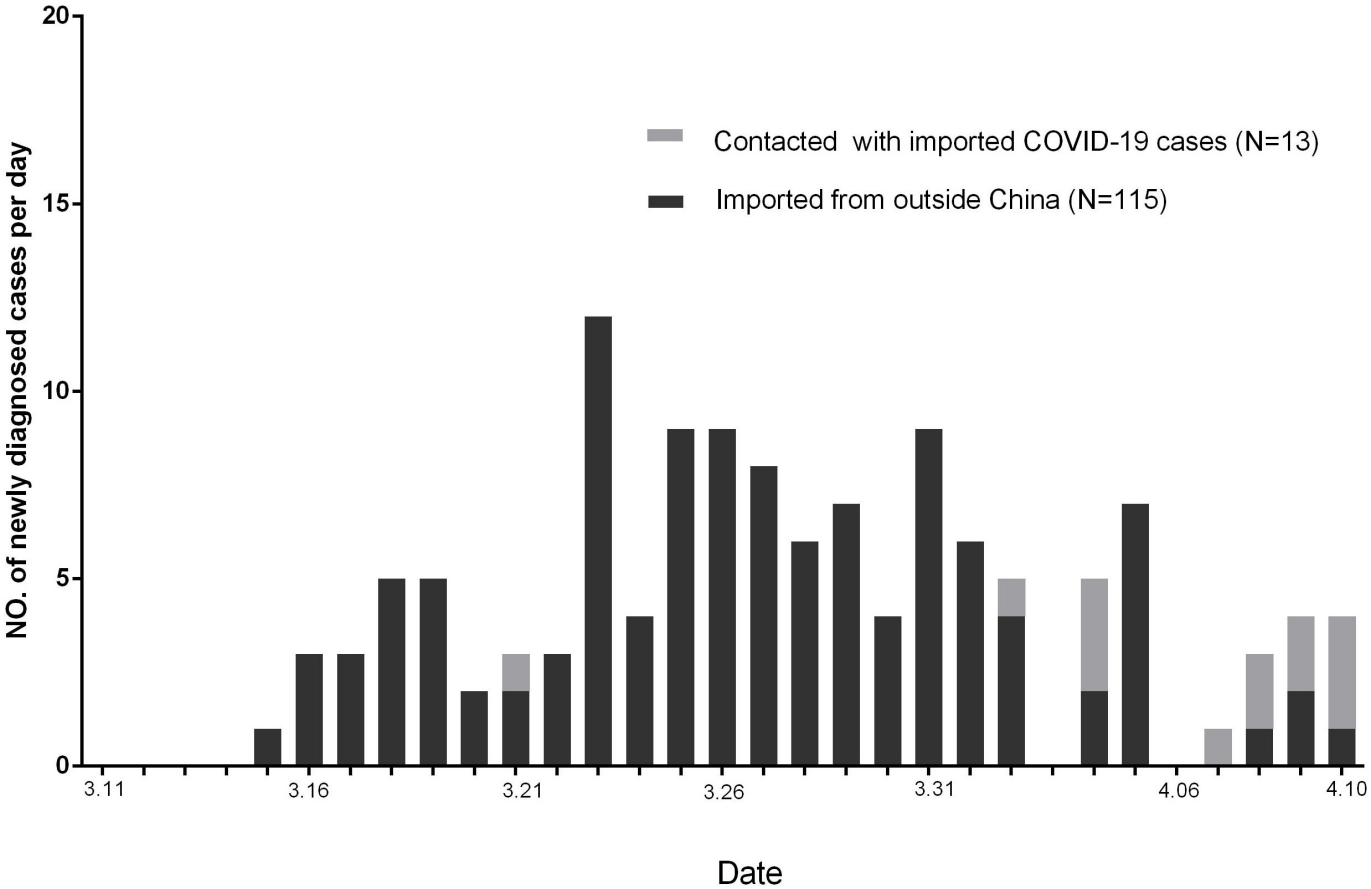
All of newly diagnosed COVID-19 cases in Guangzhou from March 11 to April 10.

Altogether, 128 of newly diagnosed COVID-19 cases in Guangzhou were reported by CDC from March 11, 2020, to April 10, 2020 (Figure 2). Among these patients, 115 were imported from outside China, 13 were with a contact history with imported COVID-19 patients from outside China, and no one were in contact with the cured patients.

We found that the patients in the IgG positive group were older (49.1 vs. 43.2, *P* = 0.031), hospitalized longer (21.0 vs. 14.0, *P* < 0.001), had more severe disease (18.2 vs. 3.0, *P* = 0.049), and with higher proportion of antibiotics treatment (88.3 vs. 63.6, *P* = 0.001) than in the negative group (Table 1). There was no difference between the two groups in terms of transmission source, incubation period, and comorbidities (all *P* > 0.05). The complications of COVID-19 included acute respiratory distress syndrome (ARDS), septic shock, acute liver failure, acute renal failure, and acute heart injury. There was no difference between the IgG positive group and negative group with regard to complications (all *P* > 0.05). No differences were found in the treatment comprised mechanical ventilation, glucocorticoids, intensive care between the two groups (all *P* > 0.05).

**Table 1:** Baseline characteristics of patients with COVID-19.

**Baseline characteristics**	**IgG positive (*N* = 154)**	**IgG negative (*N* = 33)**	**P**
Age, year	49.1 ± 14.4	43.2 ± 12.8	0.031^*^
Female Sex, *N* (%)	86 (55.8)	19 (57.6)	0.856
Incubation period, day	4.0 (8.0)	4.0 (7.0)	0.501
Interval from diagnosis to hospitalization, day	1.0 (2.3)	2.0 (3.8)	0.046^*^
Hospitalization days, day	21.0 (19.0)	14.0 (8.5)	<0.001^*^
Exposure to source of transmission			0.289
Contact with Hubei residents, *N* (%)	94 (61.0)	16 (48.5)	
Contact with COVID-19 patients, *N* (%)	38 (24.7)	9 (27.3)	
Others, *N* (%)	22 (14.3)	8 (24.2)	
Severe disease, *N* (%)	28 (18.2)	1 (3.0)	0.049^*^
Comorbidities			
Any, *N* (%)	67 (43.5)	14 (42.4)	0.999
Cardiovascular disease, *N* (%)	31 (20.1)	6 (18.2)	0.799
Diabetes, *N* (%)	7 (4.5)	4 (12.1)	0.204
Malignancy, *N* (%)	3 (1.9)	0 (0)	0.999
Chronic respiratory disease, *N* (%)	3 (1.9)	2 (6.0)	0.463
Chronic kidney disease, *N* (%)	2 (1.3)	0 (0)	0.999
Chronic liver disease, *N* (%)	7 (4.5)	3 (0.9)	0.385
Cerebrovascular disease, *N* (%)	4 (2.6)	0 (0)	0.999
White blood cell counts, 109/L	5.1 (2.3)	5.3 (3.4)	0.225
Ureanitrogen, mmol/L	3.7 (1.4)	3.6 (1.4)	0.234
Creatinine, μmol/L	60.7 (29.6)	60.0 (22.6)	0.565
Procalcitonin > 0.25 μg/L, *N* (total N)	62 (100)	7 (16)	0.167
Albumin, g/L	39.7 ± 5.7	40.6 ± 3.7	0.404
CRP > 10ng/L, *N* (total N)	59 (134)	5 (18)	0.190
ALT, U/L	25.0 (23.0)	18.9 (6.5)	0.011^*^
AST, U/L	19.3 (12.7)	16.6 (7.0)	0.008^*^
Abnormal chest CT, *N* (%)	151 (98.1)	29 (87.9)	0.183
Complications			
Any, *N* (%)	34 (22.1)	4 (12.1)	0.197
ARDS, *N* (%)	22 (14.3)	1 (3.0)	0.135
Acute cardiac injury, *N* (%)	5 (3.2)	1 (3.0)	0.999
Septic shock, *N* (%)	3 (1.9)	0 (0)	0.999
Acute kidney injury, *N* (%)	1 (0.6)	0 (0)	0.999
Acute liver injury, *N* (%)	17 (11.0)	3 (9.1)	0.777
Treatments			
Antibiotics, *N* (%)	136 (88.3)	21 (63.6)	0.001^*^
Mechanical ventilation, *N* (%)	13 (8.4)	0 (0)	0.129
Systemic glucocorticoids, *N* (%)	6 (3.2)	0 (0)	0.375
ICU Admission, *N* (%)	6 (3.9)	1 (3.0)	0.999
IgM positive, *N* (%)	35 (22.7)	0 (0)	0.001^*^

*CRP, C-reactive protein; ALT, alanine transaminase; AST, aspartate transaminase; CT, computed tomography; ARDS, acute respiratory distress syndrome; ICU, intensive care unit*.

* *P-values < 0.05. Reference range of Procalcitonin, <0.05 μg/L. Reference range of CRP, 0–10 ng/L*.

Potential variables, including age (OR, 1.03; 95% CI, 1.00–1.06; *P* = 0.033), hospitalization days (OR, 1.08; 95% CI, 1.03–1.13; *P* = 0.003), severe disease (OR, 7.11; 95% CI, 0.93–54.26; *P* = 0.058), abnormal chest CT (OR, 6.94; 95% CI, 1.48–32.67; *P* = 0.014), and antibiotics treatment (OR, 4.32; 95% CI, 1.82–10.23; *P* = 0.001), that might be associated with antibody production were screened by using univariate logistic regression analyses (Table 2). In the multivariate logistic regression model, determinants associated with antibody production comprised hospitalization days (OR: 1.06, 95%CI: 1.02–1.11, *P* = 0.006) and antibiotics treatment (OR: 3.50, 95%CI: 1.40–8.77, *P* = 0.007).

**Table 2:** Determinants associated with appearance of antibody in cured COVID-19 patients.

	**Antibody positive**		
**Determinants**	**OR**	**95% CI**	***P***
Univariate modle			
Age	1.03	1.00–1.06	0.033^*^
Hospitalization days	1.08	1.03–1.13	0.003^*^
Severe cases	7.11	0.93–54.26	0.058
Abnormal chest CT	6.94	1.48–32.67	0.014^*^
Antibiotics Treatment	4.32	1.82–10.23	0.001^*^
Multivariate modle			
Hospitalization days	1.06	1.02–1.11	0.006^*^
Antibiotics Treatment	3.50	1.40–8.77	0.007^*^

*CT, computed tomography*.

* *P-values < 0.05*.

Out of these 187 patients, 35 (18.7%) patients showed positive results and 152 (81.3%) showed negative results for IgM (Table 3). There were 154 (82.4%) patients with positive results and 33 (17.6%) patients with negative results for IgG. The antibody tests were performed after 53 days on an average from the date of disease onset. Of the 35 IgM positive cases, 12 cases turned negative during the follow up. PCR assays were undertaken in all patients using both pharyngeal and anal swabs. They yielded two positive pharyngeal swabs, seven positive anal swabs, and one positive result for both pharyngeal and anal swabs. On further retesting, all the positive results of PCR assays were found to be negative.

**Table 3:** Outcomes of cured patients with COVID-19.

**Outcomes**	**Total (*N* = 187)**	**PCR positive (*N* = 10)**	**PCR negative (*N* = 177)**	***P***
IgG positive, *N* (%)	154	8 (80.0)	146 (82.4)	0.999
IgM positive, *N* (%)	35	2 (20.0)	33 (18.6)	0.999
First antibody tests from onset, day	53.0 ± 9.9	50.3 ± 16.5	53.2 ± 9.4	0.369
Follow-up time, day	45.7 ± 11.2	48.7 ± 11.7	45.5 ± 11.1	0.380
Re-infected, *N*	0	0	0	N/A
Fever during follow-up, *N*	0	0	0	N/A
Transmission after discharge, *N*	0	0	0	N/A
Reported by the cured, *N*	0	0	0	N/A
Reported by CDC, *N*	0	0	0	N/A
Contact with newly diagnosed patients, *N*	0	0	0	N/A

*PCR, polymerase chain reaction; CDC, Centers for Disease Control and Prevention*.

In the IgG positive group, eight patients demonstrated positive results on PCR from two pharyngeal swabs and six anal swabs. In the IgG negative group, one patient had positive pharyngeal swabs and one both pharyngeal and anal swabs. We found no relationship between IgG test and PCR assay. Of the 35 IgM positive patients, two had positive anal swabs and no pharyngeal swabs. There was no relationship between IgM test and PCR assay.

## Discussion

In this retrospective observational study, we investigated the clinical features of the cured or recovered COVID-19 patients for the first time. Although they were PCR or IgM positive, these patients displayed no clinical manifestations of infection, and no signs of new acute infection were found on chest CT, indicating that these patients did not meet the re-infection criteria. Based on these findings, a positive result on PCR or IgM assay should not be considered indicative of COVID-19 re-infection. There might be several reasons for absence of re-infections. Firstly, the patients with COVID-19 were discharged from hospitals after following strict criteria, and the duration of hospital stay was more than 14 days, far exceeding that in community acquired pneumonia (10), which means that the SARS-CoV-2 was more likely to be have been eradicated. Secondly, 17.6% of the patients were negative for antibody, which might prevent a repeat infection by the virus. Thirdly, an effective prevention and control strategy ensured that the cured patients were kept away from other confirmed COVID-19 patients. Finally, the medical staffs working in the front line have not been infected till date, which effectively prevented secondary infections and spread of the disease in the hospital (11). Re-infection cases were reported in Hong Kong and the United States (12, 13). Based on the known literatures and our research, we believe that patient immunity is helpful to avoid infection, but not all patients can produce immunity after infection. Therefore, the prevention and control strategy is still the key point (14).

The antibodies can be observed in the middle and later stage of COVID-19, and performed well in the diagnosis of COVID-19 (7, 8, 15). For those who have recovered, the clinical significance of the PCR and antibody tests has not been clarified. Our study found that was resurgence of positive results of PCR or IgM tests in some patients after being discharged home. Among people who were in contact with the cured patients, no one was diagnosed with COVID-19 as reported by the Guangzhou CDC. The incubation period of COVID-19 is 3–14 days, and our follow-up period for cured patients was more than 14 days. This might have helped in excluding the cases in the incubation period of the infection. Based on these findings, we believe that the cured patients cannot cause person-to-person transmission. They also indicate that a positive result of the PCR or IgM assay does not mean that the cured patient is infectious.

IgG antibodies usually appears 3–40 days after the onset of symptoms (8). In our study, 82.4% patients produced IgG antibodies. However, IgG antibodies were not detected in 17.6% patients when tested after 53 days on an average from the onset of the disease, which means that these patients might not produce IgG antibodies. IgM antibodies appeared in 35 patients when tested after 53 days on an average following the onset of symptoms, and disappeared in 12 patients during the follow up period. Therefore, IgM antibodies might be present in some COVID-19 patients for a long time.

All COVID-19 patients were discharged home after they had negative PCR test results on two consecutive occasions, 24 h apart. However, positive results of PCR or IgM were again observed in some patients during the follow up period. The positive PCR turned to negative in the subsequent retest. Current research has not been able to explain the cause of the positive PCR retests, or confirm whether it is caused by a virus residue. Interestingly, the percentage of positive anal swabs in the cured patients was much higher than the positive pharyngeal swabs. PCR positivity of anal swabs was reported in several studies, which has led to a discussion on the possibility of fecal-oral transmission (16, 17). The reason for PCR positive anal swabs may be that the virus enters the digestive tract from the patient's mouth. However, whether the virus remains active is unknown. During the follow-up, we did not find any new confirmed COVID-19 cases that came into contact with the cured patients who demonstrated positive PCR test results from anal swabs. Although PCR positive, fecal-oral transmission could not be confirmed in our study, and further research is needed.

Compared with the IgG negative group, the IgG positive group patients were older, with longer hospital stay, higher proportion of antibiotic use, higher proportion of severe cases, and higher proportion of CT abnormalities. Further logistic regression analysis showed that the treatment of antibiotic and length of stay were risk factors for antibody production. The mechanism of antibody production associated with antibiotic treatment and long-term hospitalization is not clear. Although diabetes, cancer, and other diseases may cause a decline in immunity, they do not affect the production of antibodies. Similarly, although the use of glucocorticoids may inhibit the immune system, it also has no effect on the production of antibodies.

Studies found that IgG and IgM have a good diagnostic value in the middle and later stage of the disease (6–8, 15). However, the value of IgG and IgM in the diagnosis of cured COVID-19 patients is not clear. In our study, we found that both IgM and IgG have no relationship with PCR. Therefore, for the cured patients, IgG and IgM neither have a diagnostic value, nor can they be used to replace the PCR test. Since neither re-infection nor person-to-person transmission was found in the cured patients, IgG and IgM cannot be used to guide the prevention and control of COVID-19.

This study has the following limitations. Firstly, since this was an observational study, no interventions such as re-exposure of the cured patients to SARS-CoV-2 were performed. Therefore, it is hard to judge whether the cured patients were immune to the virus. Secondly, at the beginning of COVID-19 outbreak, there is no effective antibodies test, and the testing of antibodies were not performed at that time. So we could not compare the levels of antibody between hospitalization and follow-up. Thirdly, this was a single center study carried out in Guangzhou, a mild epidemic area. Accordingly, the conclusions of this study might not be suitable for extrapolation to other areas. Fourthly, our conclusions were based on a small sample size, which need to be further verified in a study with a large sample size. Nevertheless, our study results clarified some clinical features of the cured patients and maybe be of considerable importance for the prevention and control of COVID-19.

## Conclusions

In our study, no evidence of person-to-person transmission was found in cured COVID-19 patients. There seemed to be no re-infection in the cured COVID-19 patients in Guangzhou. These finding suggest that the cured do not cause the spread of disease. Additionally, neither IgG nor IgM can be used to replace the PCR test in cured patients.

## Data Availability Statement

The original contributions presented in the study are included in the article/supplementary materials, further inquiries can be directed to the Corresponding authors.

## Ethics Statement

The studies involving human participants were reviewed and approved by the ethics committee of the Guangzhou Eighth People's Hospital (No. 202001134). Written informed consent for participation was not required for this study in accordance with the national legislation and the institutional requirements.

## Author Contributions

GX, FL, MY, JZ, QL, YT, and WM: study concept and design. YL, HS, and FZ: acquisition of data and patient recruitment. CF and YH: analysis and interpretation of data. GX, FL, MY, JZ, and QL: drafting of the manuscript. YT and WM: revising the manuscript. All authors contributed to the article and approved the submitted version.

## Conflict of Interest

The authors declare that the research was conducted in the absence of any commercial or financial relationships that could be construed as a potential conflict of interest.
